# Genome-wide identification and expression analysis of the plant-specific PLATZ gene family in Tartary buckwheat (*Fagopyrum tataricum*)

**DOI:** 10.1186/s12870-022-03546-4

**Published:** 2022-04-01

**Authors:** Jing Li, Shan Feng, Yuchuan Zhang, Lei Xu, Yan Luo, Yuhao Yuan, Qinghua Yang, Baili Feng

**Affiliations:** 1 State Key Laboratory of Crop Stress Biology for Arid Areas / College of Agronomy, Northwest A & F University, Yangling, 712100 Shaanxi China; 2 School of Mathematics and Statistics, Northwestern Polytechnical University, Xi’an, 710129 Shaanxi China

**Keywords:** Phylogenetic analysis, Tandem duplication, Segmental duplication, Synteny analysis, *cis*-acting element, Exogenous hormones

## Abstract

**Background:**

Plant AT-rich sequence and zinc-binding (PLATZ) proteins belong to a novel class of plant-specific zinc-finger-dependent DNA-binding proteins that play essential roles in plant growth and development. Although the PLATZ gene family has been identified in several species, systematic identification and characterization of this gene family has not yet been carried out for Tartary buckwheat, which is an important medicinal and edible crop with high nutritional value. The recent completion of Tartary buckwheat genome sequencing has laid the foundation for this study.

**Results:**

A total of 14 *FtPLATZ* proteins were identified in Tartary buckwheat and were classified into four phylogenetic groups. The gene structure and motif composition were similar within the same group, and evident distinctions among different groups were detected. Gene duplication, particularly segmental duplication, was the main driving force in the evolution of *FtPLATZs*. Synteny analysis revealed that Tartary buckwheat shares more orthologous PLATZ genes with dicotyledons, particularly soybean. In addition, the expression of *FtPLATZs* in different tissues and developmental stages of grains showed evident specificity and preference. *FtPLATZ3* may be involved in the regulation of grain size, and *FtPLATZ4* and *FtPLATZ11* may participate in root development. Abundant and variable hormone-responsive *cis*-acting elements were distributed in the promoter regions of *FtPLATZs*, and almost all *FtPLATZs* were significantly regulated after exogenous hormone treatments, particularly methyl jasmonate treatment. Moreover, *FtPLATZ6* was significantly upregulated under all exogenous hormone treatments, which may indicate that this gene plays a critical role in the hormone response of Tartary buckwheat.

**Conclusions:**

This study lays a foundation for further exploration of the function of *FtPLATZ* proteins and their roles in the growth and development of Tartary buckwheat and contributes to the genetic improvement of Tartary buckwheat.

**Supplementary Information:**

The online version contains supplementary material available at 10.1186/s12870-022-03546-4.

## Background

Transcription factors (TFs) are sequence-specific binding proteins that can activate or inhibit the expression of target genes by recognizing and binding to *cis*-acting elements in their promoter regions of target genes to affect diverse biological processes at the transcriptional level [1]. Zinc finger proteins are an important class of TFs. Based on previous reports, more than 1500 TFs exist in *Arabidopsis*, accounting for approximately 5% of the *Arabidopsis* genome [2], of which approximately 15% are zinc finger proteins [3]. The zinc-finger protein consists of two cysteines and two histidines tetrahedrally coordinated with zinc atoms to form a compact finger-like structure. These proteins participate extensively in plant growth and development and actively respond to various stresses [3, 4]. Plant AT-rich sequence and zinc-binding (PLATZ) proteins are a novel class of plant-specific zinc-dependent DNA-binding proteins that preserve the unique structure of the zinc-finger protein family and contain two distantly conserved domains: C-x_2_-H-x_11_-C-x_2_-C-x_(4–5)_-C-x_2_-C-x_(3–7)_-H-x_2_-H, and C-x_2_-C-x_(10–11)_-C-x_3_-C [5]. Although the first PLATZ gene, *PLATZ1*, was isolated from peas in 2001 [5], it has attracted increasing attention from researchers. *PLATZ1* can nonspecifically bind to A/T-rich sequences and inhibit transcription, as demonstrated by a transient assay [5]. Previous studies have shown that PLATZ proteins play essential roles in several biological processes in plants. For example, Li et al. reported that *Floury3* (*FL3*) encodes a PLATZ protein in maize, which interacts with RNA polymerase III subunit 53 (RPC53) and transcription factor class C 1 (TFC1) to affect endosperm development and filling in seeds [6]. *GL6*, a PLATZ protein in rice, has been demonstrated to regulate grain length and spikelet number through the same interaction mechanism [7]. *SHORT GRAIN6* (*SG6*) regulates the division of spikelet hull cells and determines seed size in rice by interacting with DP proteins and cell division regulators [8]. Plant regulation by PLATZ is not restricted to the seeds. In *Arabidopsis*, the PLATZ protein *ORESARA15* (*ORE15*) could regulate leaf growth and senescence by promoting the rate and duration of early cell proliferation [9]. *ABA-INDUCED expression 1* (*AIN1*) represses the elongation of the primary root of *Arabidopsis* upon ABA induction [10]. In addition, Chao et al. illustrated via transcriptome analysis that PLATZ TFs are important for the secondary growth of *Populus* stems [11]. Moreover, PLATZ proteins are extensively involved in the response to numerous abiotic stresses, including heat [12], drought [13, 14], salt and osmotic stresses [15, 16], and in response to hormones [17, 18].

Tartary buckwheat (*Fagopyrum tataricum*, *2n* = *2x* = 16) is primarily cultivated in Asia, Europe and North America [19]. As a traditional medicinal and edible crop, its grain has a balanced essential amino acid composition and is rich in phytochemicals and soluble fiber [20]. In particular, flavonoids, which have many important biomedical functions, are more abundant in Tartary buckwheat than in other main crops [21–24]. Tartary buckwheat has been recognized as a green food for humans in the twenty-first century, and has gained popularity among consumers. However, its low yield severely limits its industrial applications [25]. Therefore, identifying PLATZ proteins in Tartary buckwheat is necessary because of their functional potential, particularly their regulatory roles in the growth and development of plant seeds and their relationship to plant resistance, which could provide new insights into the yield improvement of Tartary buckwheat. The PLATZ family has been identified in several other plant species. To date, 12 members have been identified in *Arabidopsis thaliana* [26], 15 in *Oryza sativa* [26], 17 in *Zea mays* [26], 62 in *Triticum aestivum* [1] and 24 in *Brassica rapa* [27]. However, to the best of our knowledge, identification and functional characterization of the PLATZ gene family in Tartary buckwheat have not yet been reported. High-quality, chromosome-scale genome sequencing of Tartary buckwheat has recently been completed [28], laying the foundation for a systematic genome-wide study of the PLATZ gene family in Tartary buckwheat. In the present study, 14 PLATZ proteins were identified in Tartary buckwheat genome. We investigated the evolutionary relationships of *FtPLATZs* together with a comprehensive study of gene structures, conserved motif composition, and *cis*-acting elements in the promoter regions of *FtPLATZs*. Gene duplication events and their syntenic relationships with the six representative species were investigated. For functional characterization, we examined the expression profiles of *FtPLATZs* in different tissues of Tartary buckwheat and in grains at different developmental stages using real-time quantitative polymerase chain reaction (qRT-PCR). In addition, the responses of *FtPLATZs* to various exogenous hormones were investigated. This study aimed to form a foundation for further exploration of the functional mechanisms of *FtPLATZs* and contribute to the improvement of plant varieties and innovation of the germplasm in Tartary buckwheat.

## Results

### Identification of *FtPLATZ* proteins in Tartary buckwheat

**Fig. 1:**
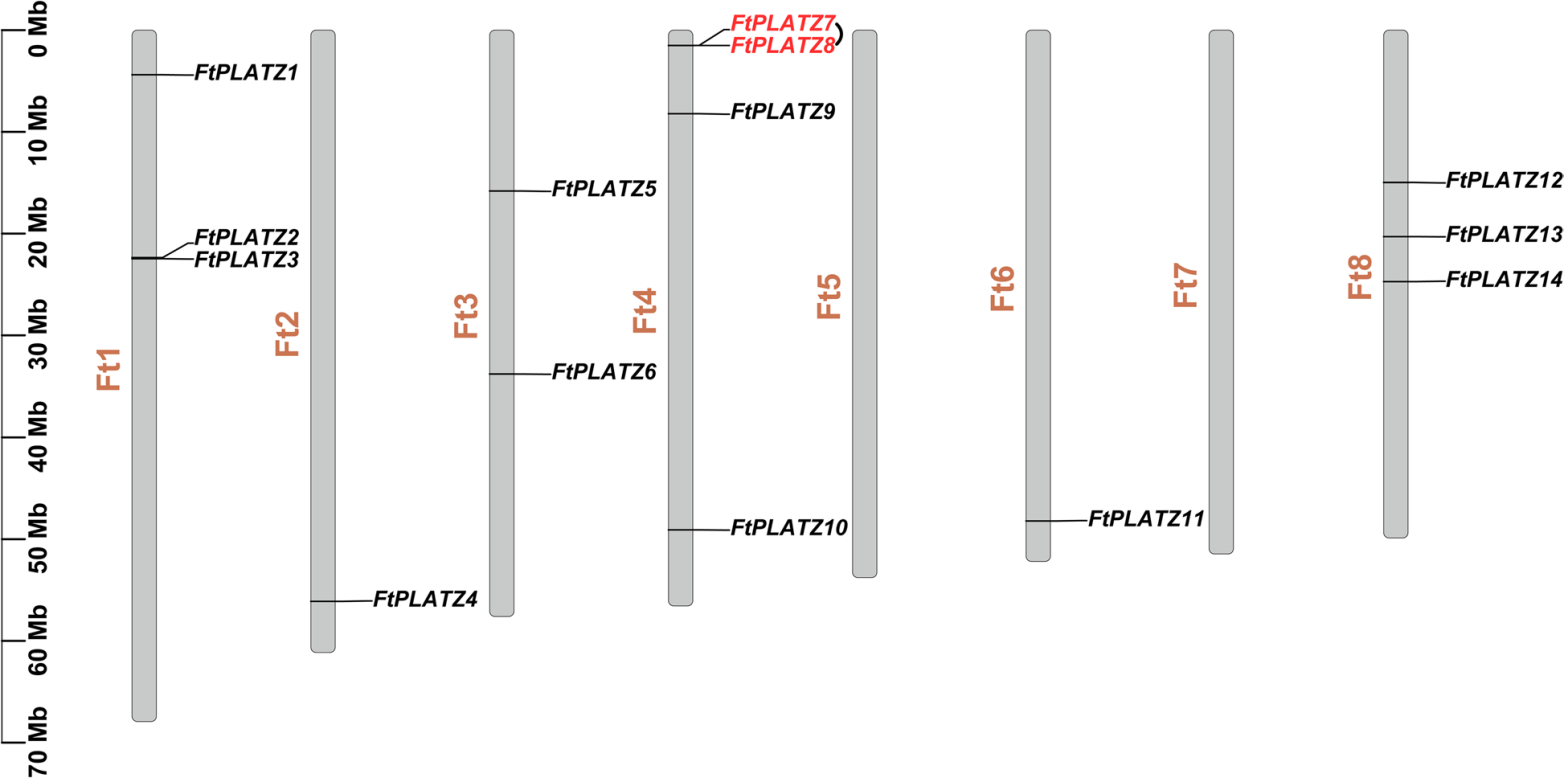
Schematic diagram of chromosomal distribution of *FtPLATZ* genes in Tartary buckwheat. The vertical bars represent the chromosomes of Tartary buckwheat, and the scale for chromosome length is shown on the left. The genes marked in red represent tandem duplication events

Combining the results of the hidden Markov model (HMM) search and BLASTP operations and further examination of the conserved PLATZ domain, 14 putative *FtPLATZ* proteins were identified in Tartary buckwheat (Fig. S1 and Table S1). They were unevenly distributed on six chromosomes of Tartary buckwheat (Fig. 1). Chromosome Ft4 contained the largest number of *FtPLATZ* genes (four genes), followed by Ft1 and Ft8, both of which contained three genes. Ft3 contained two genes, whereas Ft2 and Ft6 contained only one. The *FtPLATZ* genes were not found on chromosomes Ft5 and Ft7. We designated these as *FtPLATZ1* to *FtPLATZ14* based on their location on the chromosomes. As shown in Table 1, the full-length cDNAs, predicted protein products and Mw of *FtPLATZ* genes varied greatly, ranging from 444 to 1590 bp, 148 to 530 aa, and 16.59 to 58.82 kDa, respectively. The average coding sequence (CDS) length, predicted protein products, and molecular weight (Mw) were 805 bp, 268 aa, and 30.20 kDa, respectively. The data clearly showed that *FtPLATZ3* was the smallest, and *FtPLATZ10* exhibited the largest size with the maximum level of CDS length, predicted protein products, and Mw among *FtPLATZs*. The difference in the theoretical isoelectric point (*p*I) values among *FtPLATZ* genes was relatively small, with an average of 8.80.

**Table 1:** PLATZ family genes in Tartary buckwheat

Gene name	Gene ID	Chr location	CDS length (bp)	Protein length (aa)	Mw (kDa)	***p***I	Subcellular location
*FtPLATZ1*	FtPinG0000274100.01.T01	Ft1:4,385,017–4,386,357	825	275	30.27	8.94	Extracellular / Nucleus
*FtPLATZ2*	FtPinG0008601100.01.T01	Ft1:22,356,858–22,357,811	570	190	21.69	8.74	Extracellular / Nucleus
*FtPLATZ3*	FtPinG0001007100.01.T01	Ft1:22,468,207–22,469,090	444	148	16.59	8.53	Nucleus
*FtPLATZ4*	FtPinG0006874100.01.T01	Ft2:56,114,937–56,116,683	759	253	29.02	8.50	Nucleus
*FtPLATZ5*	FtPinG0008634800.01.T01	Ft3:15,812,380–15,813,991	657	219	24.77	9.20	Nucleus
*FtPLATZ6*	FtPinG0004423200.01.T01	Ft3:33,803,942–33,805,541	657	219	24.76	9.03	Nucleus
*FtPLATZ7*	FtPinG0009347200.01.T01	Ft4:1,503,405–1,505,568	1335	445	51.02	7.87	Nucleus
*FtPLATZ8*	FtPinG0009347000.01.T01	Ft4:1,506,059–1,508,161	1026	342	39.06	8.62	Nucleus
*FtPLATZ9*	FtPinG0008152800.01.T01	Ft4:8,214,669–8,216,255	660	220	24.61	9.03	Nucleus
*FtPLATZ10*	FtPinG0008105600.01.T01	Ft4:49,083,975–49,086,486	1590	530	58.82	8.94	Nucleus
*FtPLATZ11*	FtPinG0003450300.01.T01	Ft6:48,211,468–48,212,805	765	255	28.92	8.46	Nucleus
*FtPLATZ12*	FtPinG0008259200.01.T01	Ft8:14,969,850–14,970,983	573	191	21.12	9.47	Extracellular / Nucleus
*FtPLATZ13*	FtPinG0004797000.01.T01	Ft8:20,296,531–20,297,821	732	244	27.29	8.34	Nucleus
*FtPLATZ14*	FtPinG0002652500.01.T01	Ft8:24,723,114–24,724,351	672	224	24.89	9.50	Nucleus

*Chr* chromosome, *CDS* coding sequence, *bp* base pair, *aa* amino acid, *Mw* molecular weight, *pI* isoelectric point

### Phylogenetic analysis and classification of *FtPLATZ* proteins

**Fig. 2:**
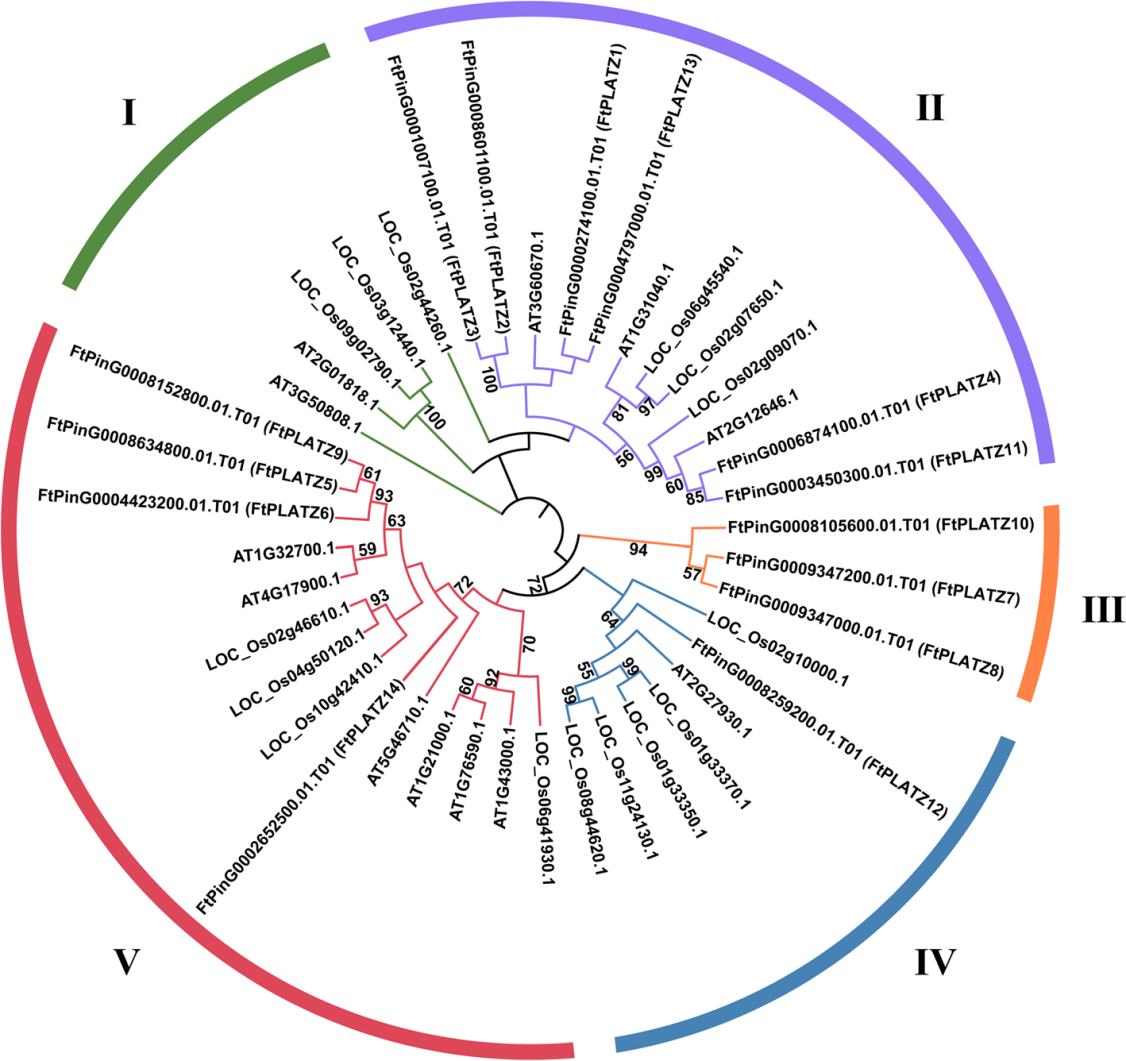
Unrooted phylogenetic tree constructed by the maximum likelihood method for PLATZ genes of Tartary buckwheat, *Arabidopsis* and rice

**Fig. 3:**
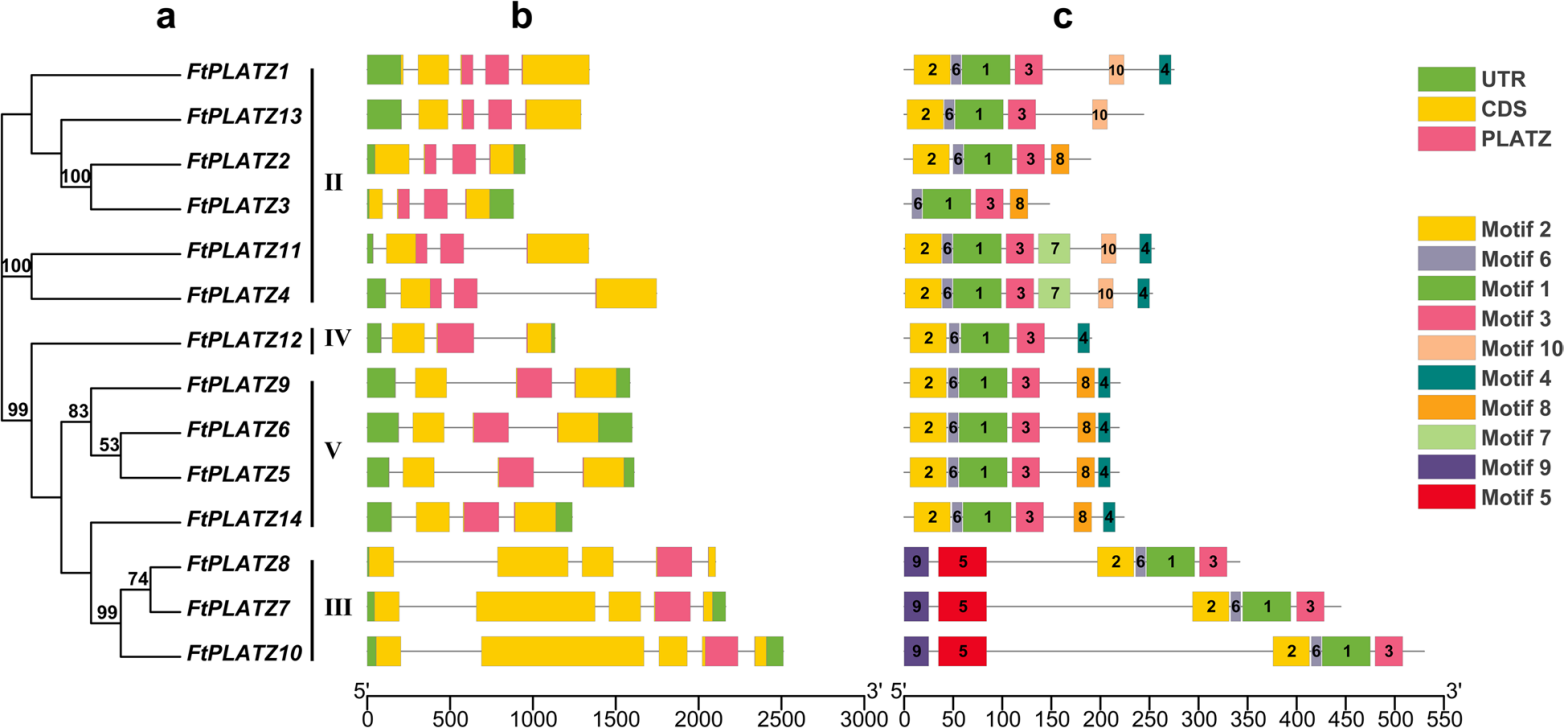
Phylogenetic relationship, gene structure and motif composition of *FtPLATZ* genes of Tartary buckwheat. **a.** Phylogenetic tree for PLATZ genes of Tartary buckwheat was constructed using the maximum likelihood method. **b.** Exon–intron structure of *FtPLATZ* genes. The legend is shown in the upper-right corner, and the introns are represented by grey lines. **c.** Motif composition of *FtPLATZ* genes. Different motifs are represented by different colors, as indicated in the legend on the right

To clarify the evolutionary relationship between the PLATZ proteins of Tartary buckwheat and the PLATZ proteins of two model plants, *Arabidopsis* and rice, we constructed a maximum likelihood (ML) tree with the 14 identified *FtPLATZs*, 12 *AtPLATZs* and 15 *OsPLATZs* (Fig. 2). The 41 PLATZ proteins were divided into five groups (I to V), and the *FtPLATZ* proteins were distributed in the four main groups (II to V). Group II contained the largest number of *FtPLATZ* members (6 of 14, 42.86%). Half of the PLATZ proteins in Group II were *FtPLATZs*. Group V contained four *FtPLATZ*s, whereas the remaining 10 proteins were from *Arabidopsis* and rice. Group IV contained one *FtPLATZ*, one *AtPLATZ* member, and five *OsPLATZ*s. In particular, group III was only composed of three *FtPLATZ* members, indicating no homology to *AtPLATZs* and *OsPLATZs.* Group I contained two *AtPLATZs* and three *OsPLATZs* but no *FtPLATZ* proteins. In addition, a phylogenetic tree for *FtPLATZs* was constructed and labeled based on the grouping in the overall phylogenetic tree to analyze the differences in gene structure and motif components among groups (Fig. 3a).

### Gene structure and conserved motifs analysis of *FtPLATZ* genes

The exon–intron structure of the *FtPLATZ* genes was investigated based on the genomic DNA sequence of Tartary buckwheat to understand the structural composition of the *FtPLATZ* genes (Fig. 3b). In general, the structures of *FtPLATZ* genes were distinguishable among the phylogenetic groups, and they showed similar characteristics within the groups. Most genes contained three introns (9 out of 14, 64.29%), and only five genes, *FtPLATZ1/7/8/10/13*, contained four introns. Group III was characterized by *FtPLATZ* genes with four introns, whereas groups IV and V contained only three-intron genes. In Group II, two genes, *FtPLATZ1* and *FtPLATZ13*, had four introns, whereas the other genes had three introns.

Result similar to the exon–intron structure was also found in the motif composition of phylogenetically grouped *FtPLATZ* members (Table S2). As shown in Fig. 3c, motifs 1, 2, 3, and 6, which constituted the core domain of PLATZ, were universally present in the *FtPLATZ* members, except for one gene (*FtPLATZ3*) in group II, where motif 2 was not present, indicating a possible sequence loss during evolution. In addition, motifs 5 and 9 were uniquely present in group III, and motifs 4 and 8 were uniquely present in group V. Motif 10 appeared only in group II, and motifs 4 and 8 were present separately in the five *FtPLATZs* of group II. Only one member of group IV, *FtPLATZ12*, possessed motif 4 exclusively, in addition to the core domain of PLATZ.

### Gene duplication events and synteny analysis of *FtPLATZ* genes

**Fig. 4:**
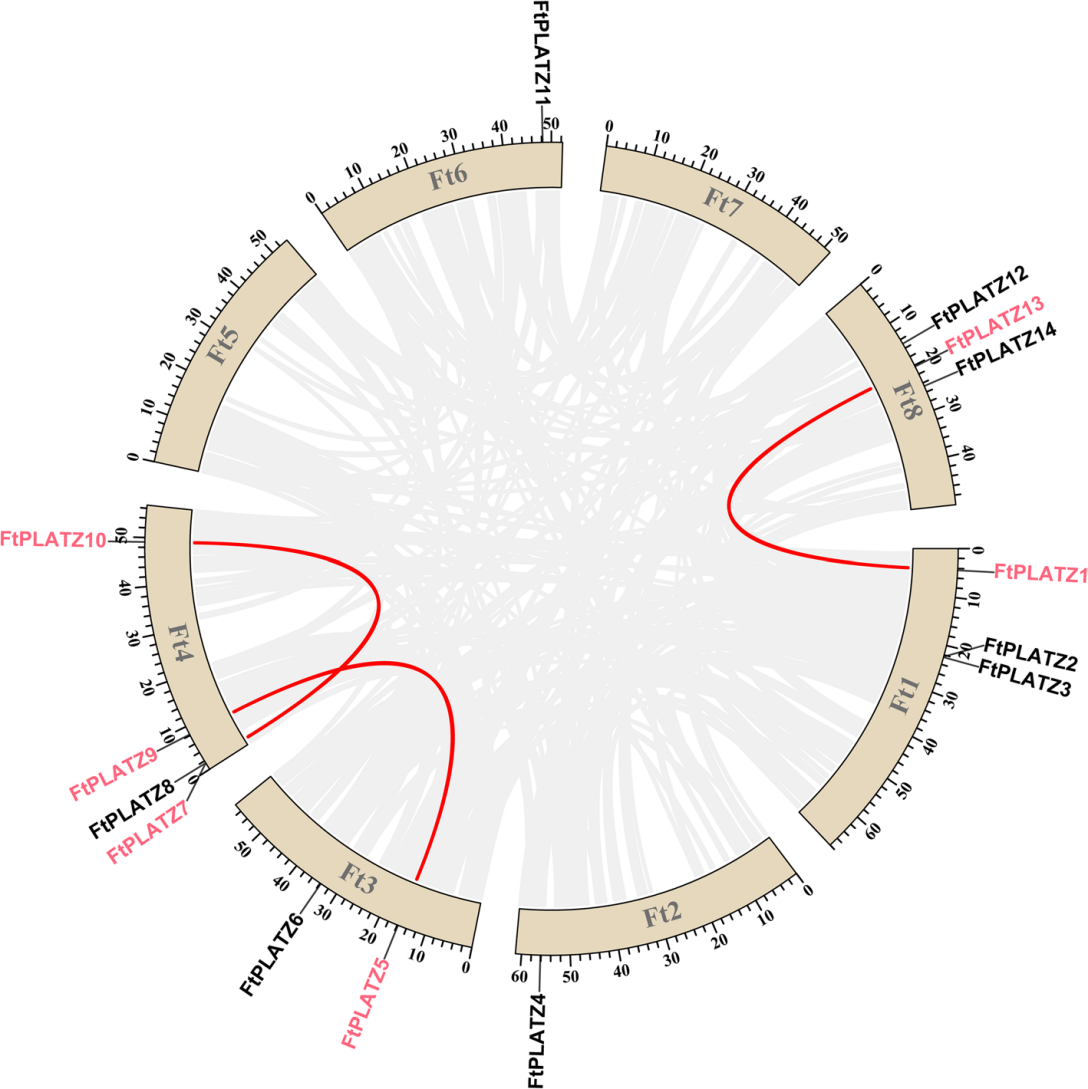
Schematic diagram of the syntenic relationships of *FtPLATZ* genes in Tartary buckwheat. The grey ribbons represent syntenic blocks in the Tartary buckwheat genome, and the segmental duplication events are marked in red

Possible gene duplication events among the *FtPLATZs* were investigated to explore the evolution of *FtPLATZ* genes. The results showed that tandem duplication and segmental duplication events were observed in *FtPLATZs*, where *FtPLATZ7*/*FtPLATZ8* formed a tandem duplication event (Fig. 1) and *FtPLATZ1/FtPLATZ13*, *FtPLATZ5*/*FtPLATZ9* and *FtPLATZ7*/*FtPLATZ10* formed three segmental duplication events (Fig. 4). These results indicate that duplication events widely participated in the evolution of *FtPLATZs*.

**Fig. 5:**
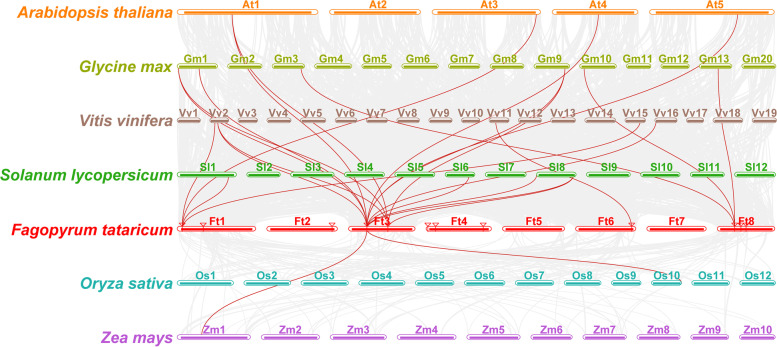
Synteny analysis of PLATZ genes between Tartary buckwheat and the other six representative plants. The syntenic gene pairs were linked by red lines

Furthermore, we investigated the syntenic relationships between *FtPLATZs* and PLATZ genes from four representative dicotyledons (*A. thaliana*, *G. max*, *V. vinifera*, and *S. lycopersicum*) and two representative monocotyledons (*O. sativa* and *Z. mays*; Fig. 5). The number of orthologous gene pairs between Tartary buckwheat and the other six species was quite different: five pairs with *Arabidopsis*, ten with soybean, six with grape, five with tomato, one with rice, and one with maize (Table S3). In particular, among the 14 *FtPLATZ* genes, *FtPLATZ5* (FtPinG0008634800.01.T01) was the only gene that was collinear with PLATZ proteins of the six representative plants. *FtPLATZ5* was collinear with at least two PLATZ genes in dicotyledons and one in monocotyledons. The results indicated that these orthologous genes may exist before the differentiation of the ancestors.

### Expression patterns of *FtPLATZ* genes in different tissues and grain developmental stages of Tartary buckwheat

**Fig. 6:**
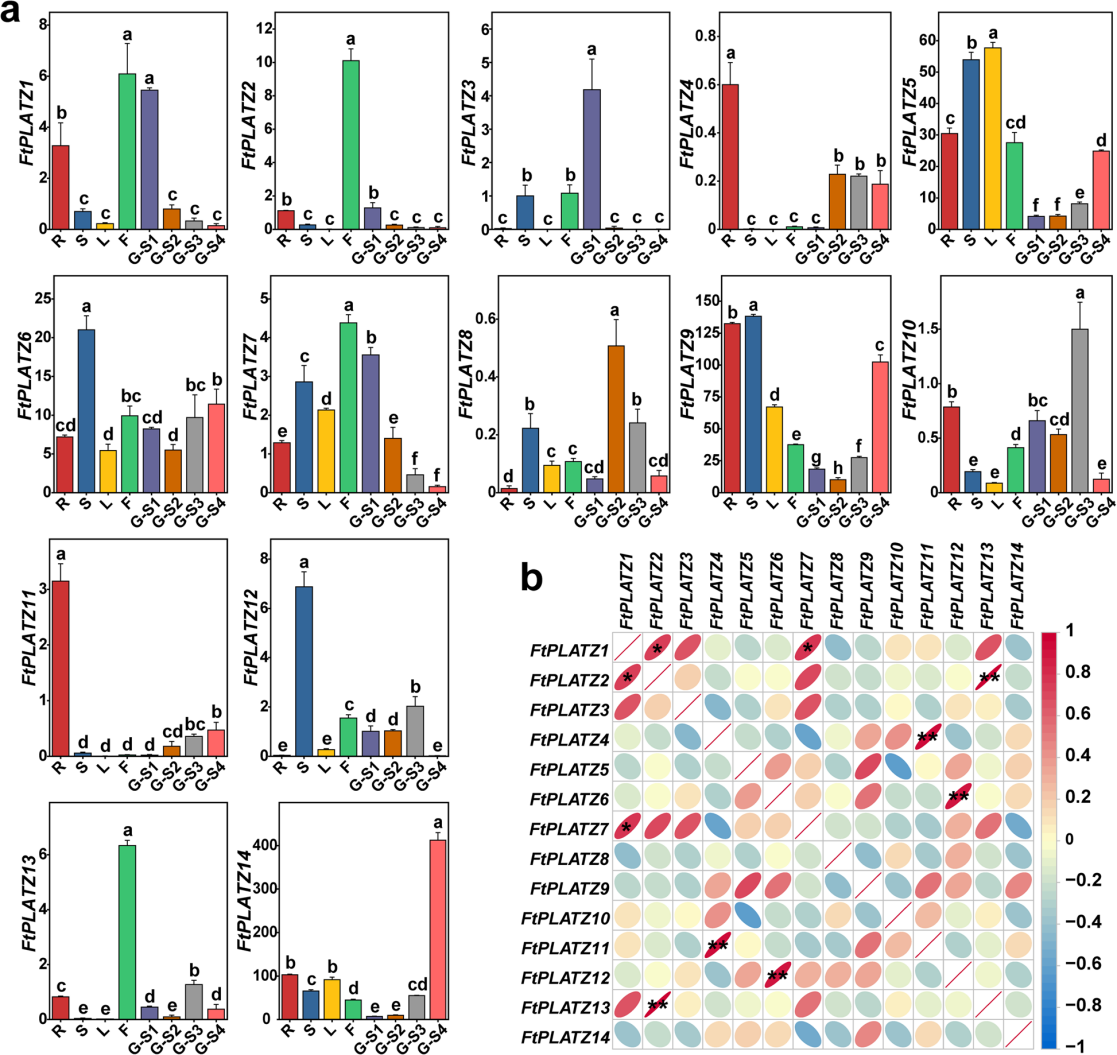
Expression profiles of *FtPLATZ* genes in different tissues and different grain developmental stages of Tartary buckwheat and correlation analysis of the expression patterns of *FtPLATZ* genes. **a.** Expression profiles of 14 *FtPLATZ* genes in the root (R), stem (S), leaf (L), flower (F), and grain (G-S1, initial formation stage; G-S2, green grain stage; G-S3, discoloration stage; G-S4, initial maturity stage) of Tartary buckwheat. Error bars are obtained from three biological replicates. Lowercase letters above the bars represent significant differences between different treatments, as determined by Duncan’s multiple range test (*p* < 0.05). **b.** Pearson’s correlation of expression patterns among *FtPLATZ* genes. Red and blue represent positive and negative correlations, respectively. * and ** indicate significance at the levels of 0.05 and 0.01, respectively

The potential roles of the identified *FtPLATZ* genes in the growth and development of Tartary buckwheat were explored using qRT-PCR (Fig. 6a and Table S4). In general, the expression patterns of *FtPLATZ* genes varied greatly in different tissues, indicating their potential multiple functions in the growth and development of Tartary buckwheat. Two genes (*FtPLATZ4* and *FtPLATZ11*) showed similar expression patterns, specifically expressed in the roots and slightly expressed in grains. Three genes (*FtPLATZ6*, *FtPLATZ9*, and *FtPLATZ12*) showed the highest expression levels in the stems. *FtPLATZ5* showed the highest expression levels in the leaves and stems. Four genes (*FtPLATZ1*, *FtPLATZ2*, *FtPLATZ7*, and *FtPLATZ13*) were highly expressed in the flowers, whereas *FtPLATZ2* and *FtPLATZ13* were only slightly expressed in other tissues. In addition, four genes (*FtPLATZ3*, *FtPLATZ8*, *FtPLATZ10*, and *FtPLATZ14*) were highly expressed in the grains, reaching their highest expression levels successively in the S1, S2, S3, and S4 developmental stages of the grains.

The expression patterns of *FtPLATZ* genes in different developmental stages of Tartary buckwheat grains have drawn much attention. Six patterns are identified. The expression levels of *FtPLATZ5*, *FtPLATZ11*, and *FtPLATZ14* increased with grain growth and development, whereas those of *FtPLATZ1*, *FtPLATZ2*, and *FtPLATZ7* decreased with grain growth and development. In addition to the two monotonous expression patterns, some gene expression levels initially decreased and then increased (*FtPLATZ6* and *FtPLATZ9*), and some initially increased and then decreased with grain development (*FtPLATZ4*, *FtPLATZ8*, and *FtPLATZ12*). In addition, the expression of *FtPLATZ10* and *FtPLATZ13* showed a wave-shaped trend, decreasing twice during stages S2 and S4 of the grains. In particular, *FtPLATZ3* was highly expressed in the S1 stage, but not in the other stages. Collectively, *FtPLATZ* genes may play crucial roles during grain development in Tartary buckwheat.

Further correlation analysis indicated that the expression patterns of some *FtPLATZ* genes in different tissues of Tartary buckwheat and different developmental stages of the grain were significantly and positively correlated (Fig. 6b); that is, *FtPLATZ1/FtPLATZ2* (*p* < 0.05), *FtPLATZ1/FtPLATZ7* (*p* < 0.05), *FtPLATZ2/FtPLATZ13* (*p* < 0.01), *FtPLATZ4/FtPLATZ11* (*p* < 0.01), and *FtPLATZ6/FtPLATZ12* (*p* < 0.01), which was consistent with the results shown in Fig. 6a, indicating that some *FtPLATZ* genes may act synergistically with one another during development.

### Analysis of promoter *cis*-acting elements of *FtPLATZ* genes

**Fig. 7:**
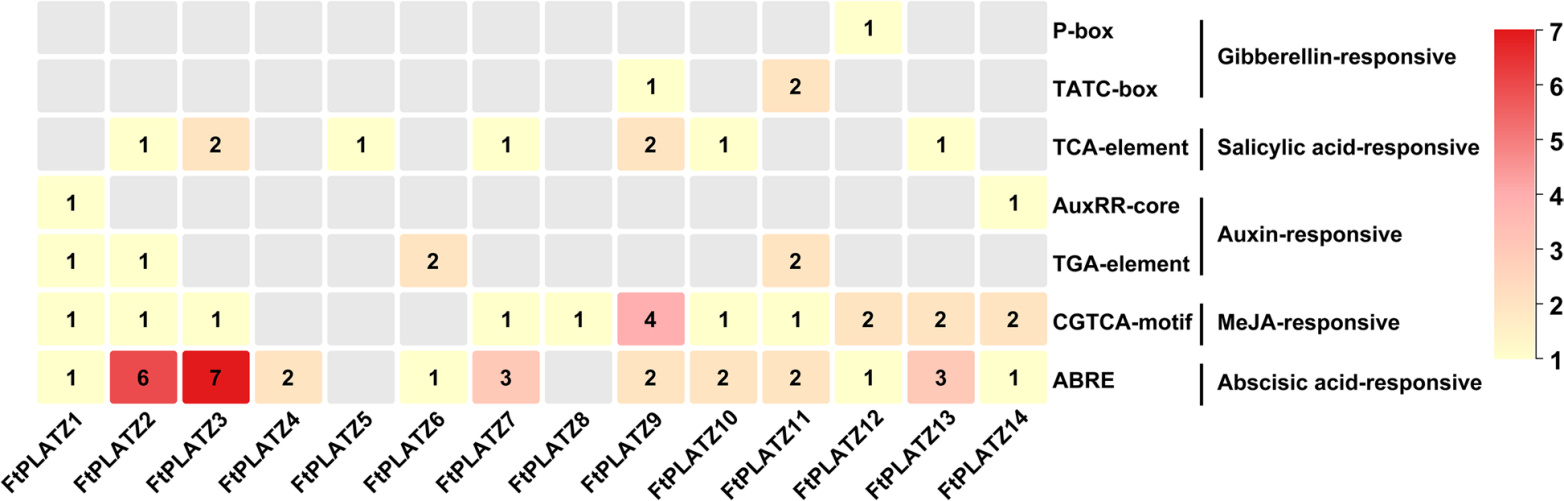
Distribution of the *cis*-acting elements related to hormone response in the promoter region of *FtPLATZ* genes

The functional potential of the identified *FtPLATZ* genes was further explored by investigating *cis*-acting elements in the promoter regions of these genes. Various *cis*-acting elements were identified, as summarized in Table S5. Promoter-related elements (i.e., TATA-box and CAAT-box) and light-responsive elements (i.e., Box 4, G-Box, TCT-motif et al.) were most abundantly distributed in the promoter region of *FtPLATZ* genes. Notably, stress-related elements (i.e., ARE, LTR, and MBS) and hormone-responsive elements (i.e., ABRE, CGTCA-motif, and TCA-element) were also widely distributed in the promoter region of the *FtPLATZ* genes. In particular, the number of hormone-responsive elements varied considerably among the *FtPLATZ* genes (Fig. 7), suggesting that the 14 *FtPLATZs* may function specifically in response to different hormone stimulation. In addition, some development-related elements (i.e., O2-site, MSA-like and CAT-box) and site-binding-related elements (i.e., CCAAT-box, HD-Zip 3 and MBSI) were identified in the promoter region of the *FtPLATZ* genes, but not all *FtPLATZs* contained such elements.

### Differential expression of *FtPLATZ* genes under different exogenous hormone treatments

**Fig. 8:**
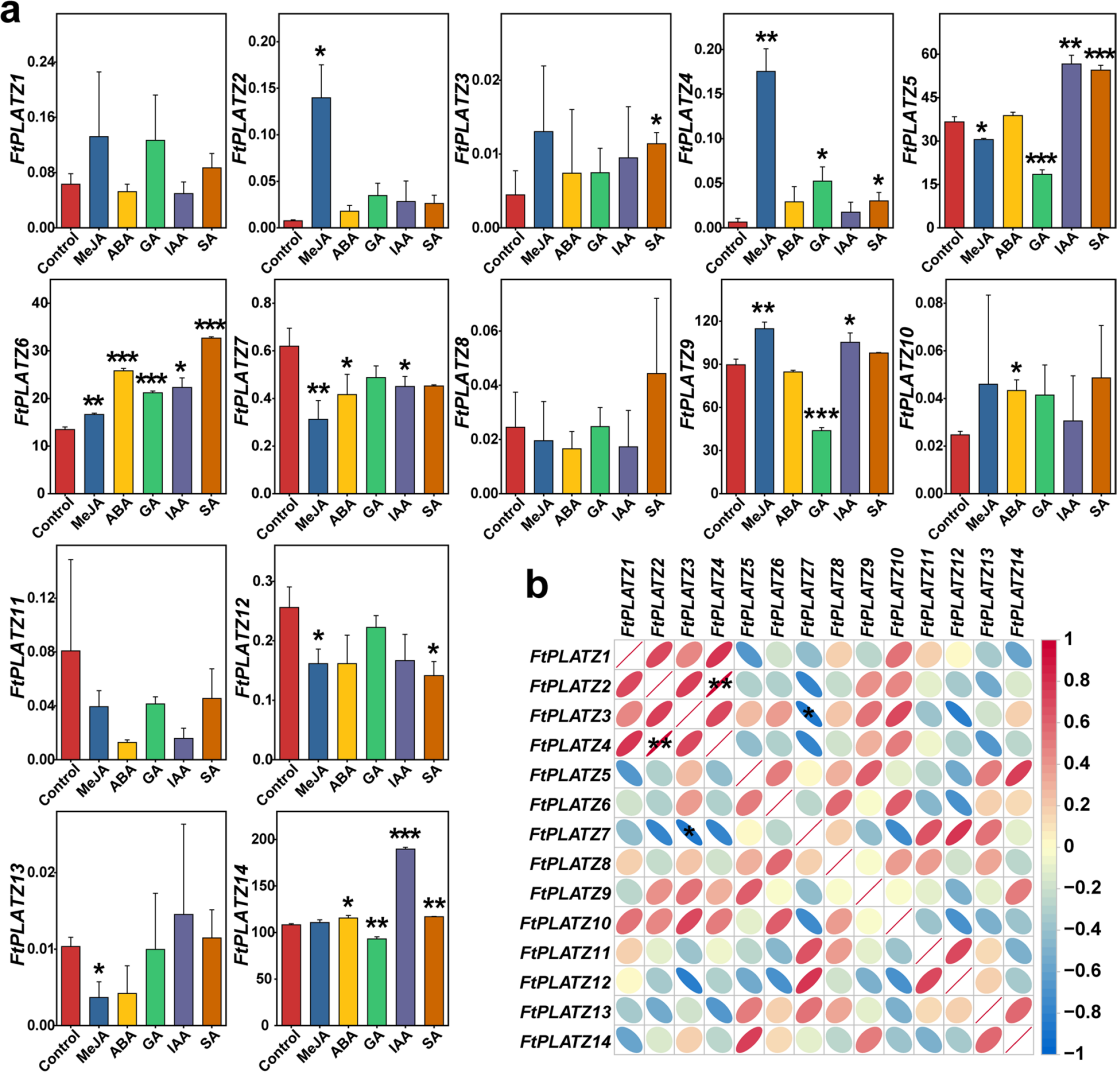
Expression profile of *FtPLATZ* genes under treatment with different exogenous hormones and their correlation analysis. **a.** Expression profiles of the 14 *FtPLATZ* genes after treatment with methyl jasmonate (MeJA), abscisic acid (ABA), gibberellin (GA), indole-3-acetic acid (IAA), and salicylic acid (SA) and the same amount of water as the control. Error bars are obtained from three biological replicates. The asterisks above the bars represent the level of significance of the expression differences under different exogenous hormone treatments compared with the control group, as determined by Student’s *t*-test. *, ** and *** indicate significance at the levels of 0.05, 0.01 and 0.001, respectively. **b.** Pearson’s correlation of response patterns to exogenous hormones among 14 *FtPLATZ* genes. Red and blue represent positive and negative correlations, respectively. * and ** indicate significance at the levels of 0.05 and 0.01, respectively

The expression levels of the 14 identified *FtPLATZ* genes after treatment with five exogenous hormones and the control, were compared using qRT-PCR to investigate the response pattern of *FtPLATZ* genes to hormones (Fig. 8a and Table S6). The results showed that the expression levels of 11 of the 14 *FtPLATZ* genes were altered significantly after treatment with at least one type of exogenous hormone. MeJA treatment had the greatest impact on *FtPLATZ* genes among the five hormones, with significant upregulation of *FtPLATZ2*, *FtPLATZ4*, *FtPLATZ6*, and *FtPLATZ9* and downregulation of *FtPLATZ5*, *FtPLATZ7*, *FtPLATZ12*, and *FtPLATZ13*. *FtPLATZ* genes, such as *FtPLATZ3*, *FtPLATZ4*, *FtPLATZ5*, *FtPLATZ6*, and *FtPLATZ14*, which responded significantly to SA treatment, were primarily upregulated. Only one gene, *FtPLATZ12*, was downregulated. Similar results were found for IAA and ABA treatments, in which the genes were primarily upregulated. In addition, under GA treatment, three genes were downregulated (*FtPLATZ5*, *FtPLATZ9*, and *FtPLATZ14*), and two genes were upregulated (*FtPLATZ4* and *FtPLATZ6*). Notably, *FtPLATZ6* was significantly upregulated by all five exogenous hormones, particularly ABA, GA, and SA. Moreover, *FtPLATZ5* and *FtPLATZ14* responded significantly to four hormones, and they showed similar response patterns to GA, IAA, and SA.

Furthermore, the results of the correlation analysis showed that the expression patterns of some genes were significantly correlated after treatment with exogenous hormones (Fig. 8b). The expression patterns of *FtPLATZ2* and *FtPLATZ4* were significantly and positively correlated (*p* < 0.01), whereas *FtPLATZ3* and *FtPLATZ7* showed a significant negative correlation (*p* < 0.05).

### Subcellular localization of *FtPLATZ* proteins

**Fig. 9:**
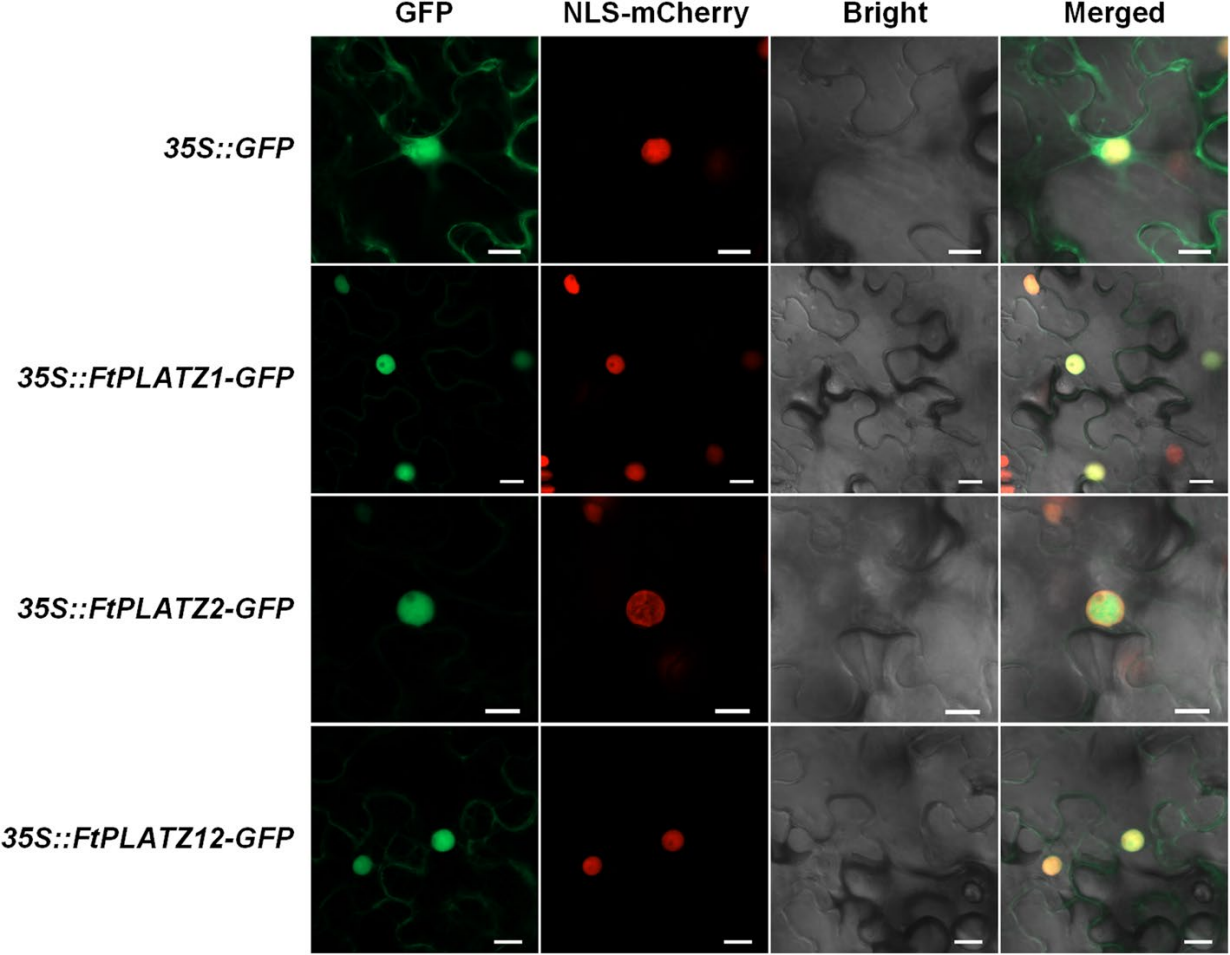
Subcellular localization of *FtPLATZ* proteins. The control (*35S::GFP*), *35S::FtPLATZ1-GFP*, *35S::FtPLATZ2-GFP*, and *35S::FtPLATZ12-GFP* fusion proteins were transiently expressed in *Nicotiana benthamiana* leaves, separately. GFP, green fluorescence of fusion proteins; NLS-mCherry, red fluorescence of the nucleus; Bright, bright field; Merged, merged microscopic images. Scale bars = 20 μm

The subcellular localization prediction results of CELLO and Plant-mPLoc consistently showed that most proteins were localized in the nucleus (11 out of 14), but the prediction results for the remaining three proteins (*FtPLATZ1*, *FtPLATZ2*, and *FtPLATZ12*) were inconsistent between the two prediction methods (Table 1). CELLO predicted that these proteins were localized extracellularly, whereas Plant-mPLoc predicted that they were located in the nucleus. Transient expression in *Nicotiana benthamiana* was examined to verify its subcellular localization (Fig. 9). These results indicated that the green fluorescent protein (GFP) fluorescent signals of the three fusion proteins were primarily localized in the nucleus. By contrast, the control *35S::GFP* signal was detected in whole cells. The experimental results suggest that *FtPLATZ* proteins may function as conventional TFs.

## Discussion

PLATZ TFs are a class of plant-specific zinc-dependent DNA-binding proteins that play important roles in the growth and development of plants and their response to stress [16]. In this study, we identified 14 PLATZ proteins in the Tartary buckwheat genome, all of which harbored conserved PLATZ domains. The amount of PLATZ proteins in Tartary buckwheat was similar to that identified in *Arabidopsis* (12) [26], rice (15) [26], and maize (17) [26]. However, the genome size of these species varied greatly (Tartary buckwheat, 489.3 Mb [28]; *Arabidopsis*, 125 Mb [29]; rice, 466 Mb [30], and maize, 2.3Gb [31]), implying that the amount of PLATZ proteins and the size of the genome were not closely related.

Gene duplication, including tandem duplication and segmental duplication, is regarded as a primary driving force in the evolution of genomes and genetic systems and is also a mechanism for organisms to adapt to changing environments [32, 33]. Fu et al. found that 21 of the 62 *TaPLATZ* genes identified in the wheat genome were from tandem duplications (33.9%) and two from segmental duplications and concluded that genomic duplication was the primary cause of the expansion of the *TaPLATZ* family [1]. Similarly, Azim et al. found a considerable number of gene duplication events in *Brassica rapa*, where 20 pairs of segmental duplication genes were detected among the 24 identified *BrPLATZ* genes, whereas no tandem duplication events were found [27]. In our study, a pair of tandem duplicated *FtPLATZ* genes (Fig. 1) and three pairs of segmental duplicated *FtPLATZ* genes (Fig. 4) were detected in Tartary buckwheat, accounting for 50% of the *FtPLATZ* genes (seven out of 14 genes), implying that gene duplication was the main driving force in the evolution of *FtPLATZ* genes. These duplicated genes had almost the same exon–intron structure and motif composition (Fig. 3b and c), but their expression preferences seemed to differ (Fig. 6a). Subfunctionalization of duplicated *FtPLATZ* genes may account for their different expression patterns [34]. Furthermore, synteny analysis showed that the *PLATZ* genes of Tartary buckwheat shared more orthologs with dicotyledons than with monocotyledons. Tartary buckwheat and soybean had the largest number of orthologous gene pairs (Table S3), implying that they could have a closer evolutionary relationship and may have evolved from a common ancestor, which conformed to previous findings [20, 35].

In the phylogenetic analysis, the 41 PLATZ proteins obtained from Tartary buckwheat, *Arabidopsis* and rice were classified into five groups based on their phylogenetic relationships, wherein 14 *FtPLATZ* proteins were distributed into four main groups (Groups II to V, Fig. 2). The exon–intron structures of *FtPLATZ* genes were similar, containing three or four introns (Fig. 3b), implying that *FtPLATZ* genes were relatively conserved during evolution [27]. *FtPLATZ* genes within the same group shared a similar gene structure and motif composition, whereas evident distinctions were found among different groups, particularly in motif composition, implying large functional differentiation of *FtPLATZ* genes. Ten motifs were detected in the *FtPLATZ* proteins, of which motifs 1, 2, 3, and 6 constituted the PLATZ domain (Fig. 3c). In our study, exon loss was observed in the *FtPLATZ* genes. In particular, *FtPLATZ3* did not contain motif 2, part of the beginning of the PLATZ domain, which may be due to genetic variation that occurred during the evolution of *FtPLATZ* genes, thereby leading to the alteration of gene functions [36]. *LOC_Os06g45540.1* (*SG6*; *GL6*) belonged to the same phylogenetic group as *FtPLATZ3* (group II), which has been proven to regulate the grain size and spikelet number of rice [7, 8]. Meanwhile, time-course transcriptome analysis revealed that *AT3G60670.1* in group II was involved in the development and maturation of *Arabidopsis* grains [37]. As shown in Fig. 6a, *FtPLATZ3* was significantly expressed at the S1 stage of grains, which is considered a critical developmental period for grain size [35]. Collectively, we hypothesized that *FtPLATZ3* may be involved in the regulation of grain size in Tartary buckwheat, and further experimental verification is necessary. In addition, *FtPLATZ4* and *FtPLATZ11* may play important roles in the development of Tartary buckwheat roots. Although they have been found to be specifically expressed in the roots through tissue expression profiles, *AT2G12646.1* (*RITF1*), located in the closest phylogenetic branch with two *FtPLATZ* genes, has been demonstrated to play a central role in mediating root meristem growth factor 1 (RGF1) signalling and subsequently affecting the size of root meristems [38].

Plant hormones play important roles in numerous biological processes and contribute remarkably to the adaptability of plants to changing environments [39, 40]. Previous studies have shown that PLATZ genes are hormone responsive. *GmPLATZ1* in soybeans [18] and PLATZ genes in *Thellungiella salsuginea* roots [17] could be induced by ABA. *GhPLATZ1* from cotton is significantly upregulated in transgenic *Arabidopsis* under ABA and GA treatments [15]. Moreover, ABA can induce the expression of *AIN1* in *Arabidopsis*, thereby affecting the elongation of the primary root [10]. *PhePLATZ* genes in moso bamboo were significantly regulated by GA, ABA, and MeJA treatments [41]. In the present study, hormone-responsive elements related to ABA, MeJA, SA, GA, and IAA were examined in the promoter region of *FtPLATZ* genes; however, their distribution across different *FtPLATZ* genes was diverse, which is similar to the findings of Fu et al. in the identification of *TaPLATZs* [1]. MeJA can activate the expression of defense genes, induce the synthesis of defensive compounds, and can also affect the antioxidant system [42]. Numerous studies have revealed that MeJA is involved in mediating defense responses against fungal pathogens [43], alleviating salt [44], drought [45], and chilling stresses [46]. The expression of nearly 80% of *FtPLATZ* genes (11 out of 14) changed significantly after treatment with exogenous hormones (Fig. 8a), among which MeJA treatment exhibited the widest effects in 8 of the 11 significantly disturbed genes, implying that *FtPLATZ* genes might be extensively involved in the stress response of Tartary buckwheat. *FtPLATZ6* was the only gene that was significantly upregulated after all exogenous hormone treatments, which may indicate the critical role of *FtPLATZ6* in biological processes involved in the hormone response of Tartary buckwheat. However, no abundant hormone-responsive elements were found in the promoter region of *FtPLATZ6* (Fig. 7). Previous studies have reported that the distribution pattern of *cis*-acting elements is not directly related to the gene expression levels [47–49]. Therefore, the expression of *FtPLATZ* genes may involve complex regulatory mechanisms that require further experimental verification.

## Conclusions

In this study, we systematically identified and characterized the PLATZ gene family in Tartary buckwheat. Fourteen *FtPLATZ* proteins were identified, which were unevenly distributed on six of the eight chromosomes in Tartary buckwheat. Based on phylogenetic analysis, the *FtPLATZ* proteins were classified into four groups, and each group shared a similar gene structure and motif composition. In addition, gene duplication, particularly segmental duplication, was the main driving force in the evolution of the *FtPLATZ* genes. We analyzed the expression levels of 14 *FtPLATZ* genes in different tissues and different grain developmental stages of Tartary buckwheat and their responses to five exogenous hormones. The results revealed, to a great extent, the important roles of *FtPLATZ* genes in the growth and development of Tartary buckwheat, such as *FtPLATZ3,* which might be involved in the regulation of grain size; *FtPLATZ4* and *FtPLATZ11*, which played a role in root development; and *FtPLATZ6,* which was significantly upregulated after all the exogenous hormone treatments and may be critical for the hormone response of Tartary buckwheat. This study provides a foundation for further exploration of the functional characteristics of *FtPLATZ* genes and promotes targeted genetic breeding research for crop improvement in Tartary buckwheat.

## Material and methods

### Identification of *FtPLATZ* genes in Tartary buckwheat genome

The Tartary buckwheat genome was obtained from the Tartary Buckwheat Genome Project (TBGP; http://www. mbkbase.org/Pinku1/), and the gene anotation V2 version was used for subsequent analysis [28]. To identify PLATZ genes in the Tartary buckwheat genome, the HMM profile of PLATZ (PF04640) downloaded from the Pfam database (http://pfam.xfam.org/) was used to search against the Tartary buckwheat genome database via HMMER3.3 with default parameter settings [50]. The PLATZ genes of *Arabidopsis* and rice obtained from the TAIR database (https://www.arabidopsis.org/) and iTAK database (http://itak.feilab.net/cgi-bin/itak/index.cgi) [51], respectively, were used to perform a BLASTP operation to further retrieve possible *FtPLATZ* genes from the Tartary buckwheat genome with a score ≥ 100 and e-value ≤1 × 10 ^− 10^ [52]. All putative *FtPLATZ* genes integrating the results of the HMM retrieval and BLASTP operations were submitted to the NCBI Conserved Domain Database (CDD, https://www.ncbi.nlm.nih.gov/cdd), SMART (http://smart.embl-heidelberg.de/), and Pfam to examine the existence of the conserved PLATZ domain.

### Sequence characterization

We collected chromosomal location information for the identified *FtPLATZ* genes from the Tartary buckwheat genome database and visualized them using TBtools [53]. The properties of the identified *FtPLATZ* genes, including CDS length, protein length, Mw, and *p*I, were investigated using Expasy (https://web.expasy.org/compute_pi/). The exon–intron structure of the *FtPLATZ* members was investigated using TBtools based on Tartary buckwheat genome annotation information. Conserved motifs of *FtPLATZ* proteins were identified using the MEME Suite (https://meme-suite.org/meme/tools/meme) with default parameters, except that the maximum number of motifs was set to 10. Moreover, the *cis*-acting elements within the 2000 bp sequence upstream of *FtPLATZ* genes, usually regarded as the promoter region of a gene [1], were analyzed using PlantCARE (http://bioinformatics.psb.ugent.be/webtools/plantcare/html/) [54].

### Phylogenetic analysis

The identified genes, together with *AtPLATZs* and *OsPLATZs*, were used to construct a phylogenetic tree using the ML method with the MEGA X software [55]. The Jones–Taylor–Thornton (JTT) model combined with a discrete gamma distribution (+ G) was selected as the optimal model for constructing the phylogenetic tree. Sequences with more than 20% alignment gaps were removed, and a bootstrap test was conducted with 1000 replicates. A phylogenetic tree containing only *FtPLATZs* was constructed using these parameters. The classification of *FtPLATZs* based on the phylogenetic tree was referred to the method described by Wang et al. [26].

### Gene duplication and synteny analysis

Possible gene duplication events among *FtPLATZ* genes were probed using multiple collinear scanning toolkits (MCScanX) [56]. Syntenic analyses were conducted using TBtools between the identified *FtPLATZ* proteins and PLATZ protein sequences of *Glycine max*, *Vitis vinifera*, *Solanum lycopersicum*, *Oryza sativa*, and *Zea mays* obtained from the iTAK database and the *AtPLATZs* obtained from the TAIR database.

### Plant materials and treatments

Weining-14, a Tartary buckwheat variety provided by the Minor Grain Crops Research Centre of Northwest A & F University, was planted in the experimental field of Northwest A & F University, Yangling, Shaanxi, China in 2020. Different Tartary buckwheat tissues, including the roots, stems, leaves, flowers, and grains at different developmental stages (3, 10, 17, and 24 days after pollination, corresponding to the initial formation stage [G_S1], green grain stage [G_S2], discoloration stage [G_S3], and initial maturity stage [G_S4], respectively) were sampled.

To investigate the response of *FtPLATZ* genes to exogenous hormones, 21-day-old seedlings (Fig. S2) were treated with different exogenous hormones, including 100 μM methyl jasmonate (MeJA), abscisic acid (ABA), salicylic acid (SA), 10 μM indole-3-acetic acid (IAA), and gibberellin (GA) by foliar spraying. The control group was sprayed with equal amounts of water. After 6 h of treatment, the second leaves of the seedlings were collected separately [57]. All the samples were collected from at least three healthy plants, immediately frozen with liquid nitrogen, and then stored at − 80 °C for RNA extraction and subsequent qRT-PCR analysis.

### Expression analyses of *FtPLATZ* genes by qRT-PCR

Total RNA was extracted from all samples using a MiniBEST Plant RNA Extraction Kit (TaKaRa). First-strand cDNA was synthesized using the PrimeScript™ II 1st Strand cDNA Synthesis Kit (TaKaRa). qRT-PCR was performed using TB Green™ *Premix Ex Taq*™ II (TaKaRa) on a Q7 Real-Time PCR System (Applied Biosystems™, Foster City, CA, USA) following the manufacturer’s instructions. The primers for qRT-PCR analysis were designed using Primer3 software (version 4.1.0, https://primer3.ut.ee/) based on the CDSs of the identified *FtPLATZ* genes obtained from TBGP, and the information of all primer sequences are listed in Table S7. *FtH3* was selected as the internal reference gene, which has been proven to be stably expressed in Tartary buckwheat under any condition [58]. Expression data were analyzed using the 2^−ΔΔC^^T^ method [59].

### Subcellular localization of *FtPLATZ* proteins

The subcellular localization of the identified *FtPLATZ* proteins was predicted using CELLO (version 2.5, http://cello.life.nctu.edu.tw/) [60] and Plant-mPLoc (version 2.0, http://www.csbio.sjtu.edu.cn/bioinf/plant-multi/) [61]. Three proteins, namely, *FtPLATZ1*, *FtPLATZ2*, and *FtPLATZ12*, with inconsistent results (CELLO predicted as extracellular, whereas Plant-mPLoc predicted as nuclear) were selected to verify the prediction of subcellular localization. The CDSs of *FtPLATZs* (excluding stop codons) were cloned from the cDNA for qRT-PCR using the primers listed in Table S7 and then inserted into the pCAMBIA2300-GFP vector driven by a 35S promoter. The recombinant plasmids *35S::FtPLATZ1-GFP*, *35S::FtPLATZ2-GFP*, and *35S::FtPLATZ12-GFP* were constructed and transformed into *Agrobacterium tumefaciens* strain GV3101 (Shanghai Weidi Biotechnology Co., Ltd., Shanghai, China). Transient expression was performed in *N. benthamiana* leaves in accordance with the method of Fu et al. [1], and the GFP fluorescence signal was detected by confocal laser scanning microscopy (LSM880; Carl Zeiss, Germany).

### Statistical analysis

Comparisons of the expression levels of the *FtPLATZ* genes in different tissues were statistically evaluated by one-way analysis of variance (ANOVA) using IBM SPSS Statistics 25 (IBM Corporation, Armonk, NY) [62]. Duncan’s multiple range test was used to determine significant differences between groups. Student’s *t*-test was carried out using R software (version 4.0.2) to examine whether the expression of *FtPLATZ* genes changed significantly after stimulation with exogenous hormones.

## Supplementary Information

**Additional file 1: Table S1.** CDS and protein sequences of *FtPLATZs* identified in this study.

**Additional file 2: Table S2.** Putative motifs identified in *FtPLATZ* proteins by MEME.

**Additional file 3: Table S3.** Orthologous gene pairs between Tartary buckwheat and other six representative species.

**Additional file 4: Table S4.** Raw data of the expression profiles of *FtPLATZ* genes in different tissues and in different grain developmental stages of Tartary buckwheat analyzed by qRT-PCR.

**Additional file 5: Table S5.** *Cis*-acting elements in the promoter regions of *FtPLATZs*.

**Additional file 6: Table S6.** Raw data of the expression profiles of *FtPLATZ* genes in response to different exogenous hormone treatments analyzed by qRT-PCR.

**Additional file 7: Table S7.** Primers of *FtPLATZ* genes used in this study.

**Additional file 8: Fig. S1.** Multiple sequence alignment of PLATZ proteins in Tartary buckwheat.

**Additional file 9: Fig. S2.** Picture of 21-day-old Tartary buckwheat seedlings treated with different exogenous hormones.

## Data Availability

The genome sequences of Tartary buckwheat used for identifying PLATZ genes in this study were located in the Tartary Buckwheat Genome Project (TBGP; http://www.mbkbase.org/Pinku1/). The Tartary buckwheat accession (Weining-14) used in the experiment was provided by the Minor Grain Crops Research Centre of Northwest A & F University. The datasets supporting the conclusions of this article are included in the article and its Supplementary files.

## Abbreviations

aa: Amino acid
ABA: Abscisic acid
AIN1: ABA-INDUCED expression 1
ANOVA: One-way analysis of variance
At: *Arabidopsis thaliana*
bp: Base pair
Br: *Brassica rapa*
CDD: Conserved Domain Database
CDS: Coding sequence
FL3: Floury3
Ft: *Fagopyrum tataricum*
GA: gibberellin
GFP: Green fluorescent proteins
Gh: *Gossypium hirtusum*
Gm: *Glycine max*
G_S1: Initial formation stage
G_S2: Green grain stage
G_S3: Discoloration stage
G_S4: Initial maturity stage
HMM: Hidden Markov model
IAA: Indole-3-acetic acid
JTT: Jones-Taylor-Thornton
MeJA: Methyl jasmonate
ML: Maximum likelihood
Mw: Molecular weight
ORE15: ORESARA15
Os: *Oryza sativa*
Phe: *Phyllostachys edulis*
*p*I: Isoelectric point
PLATZ: Plant AT-rich sequence and zinc-binding
qRT-PCR: Real-time quantitative polymerase chain reaction
RGF1: Root meristem growth factor 1
RPC53: RNA polymerase III subunit 53
SA: Salicylic acid
SG6: SHORT GRAIN6
Ta: *Triticum aestivum*
TBGP: Tartary Buckwheat Genome Project
TFC1: Transcription factor class C 1
TFs: Transcription factors
