# Supplementary figures and images for: Genome-wide identification and expression analysis of the plant-specific PLATZ gene family in Tartary buckwheat (Fagopyrum tataricum)
Source: BMC Plant Biol. 2022 Apr 1;22:160. doi: 10.1186/s12870-022-03546-4 (PMC8974209; doi:10.1186/s12870-022-03546-4)

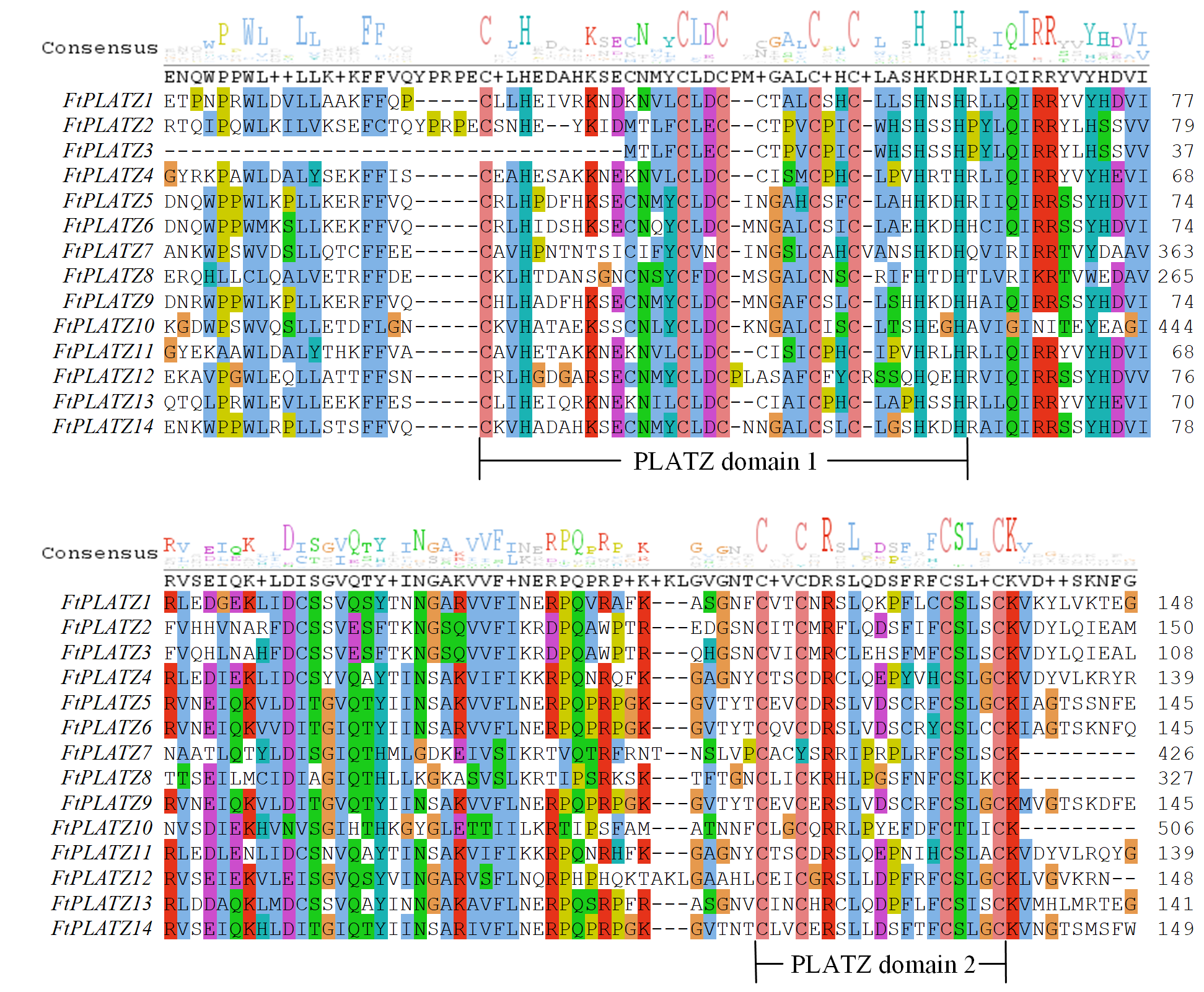

Supplement: Supplementary file 8 — Additional file 8: Fig. S1. Multiple sequence alignment of PLATZ proteins in Tartary buckwheat. [file 12870_2022_3546_MOESM8_ESM.tif]

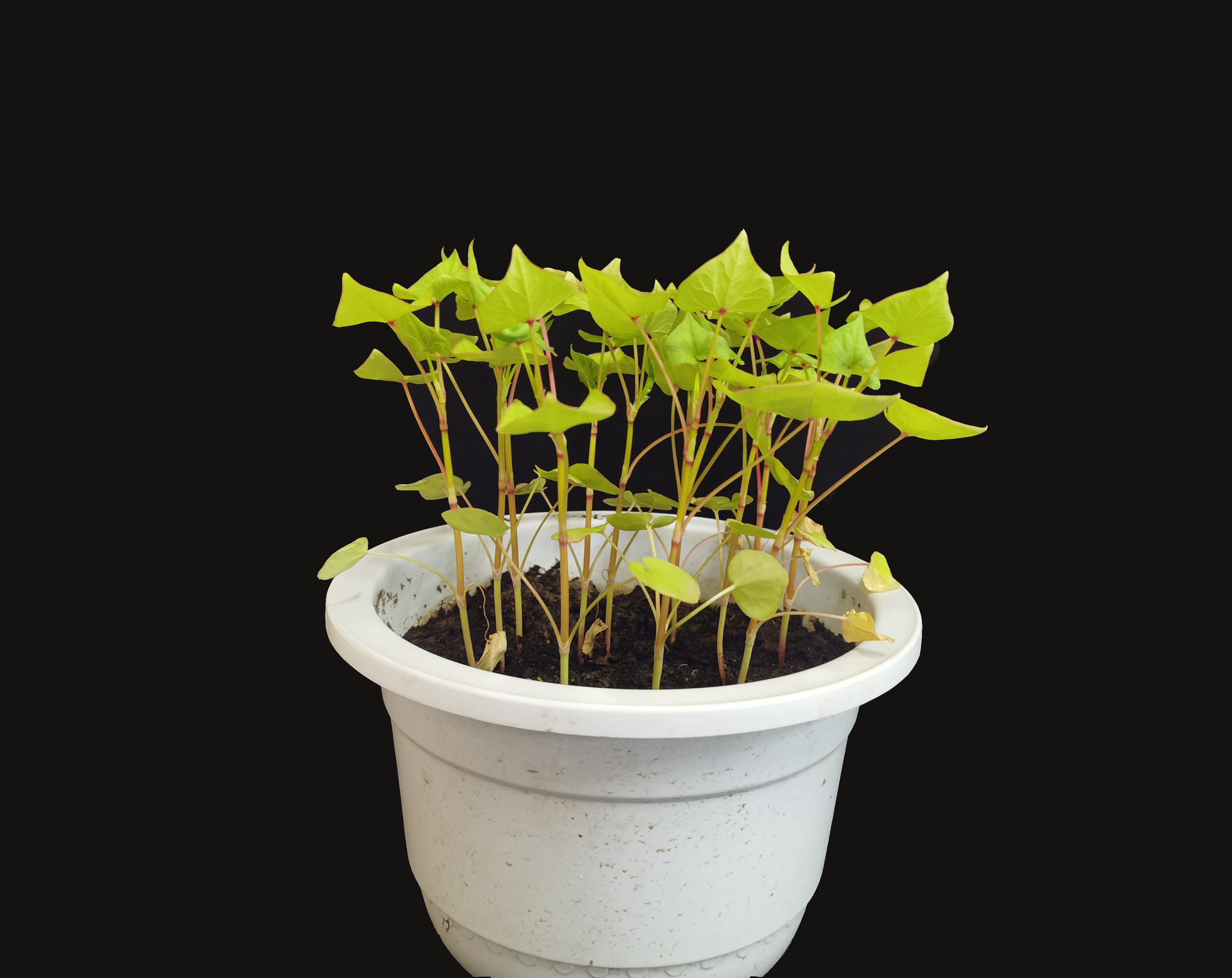

Supplement: Supplementary file 9 — Additional file 9: Fig. S2. Picture of 21-day-old Tartary buckwheat seedlings treated with different exogenous hormones. [file 12870_2022_3546_MOESM9_ESM.tif]
